# *More than one and less than many*: research and care in pediatric first-in-human cell and gene therapy clinical trials

**DOI:** 10.3389/fgene.2026.1952672

**Published:** 2026-10-14

**Authors:** Stanislav Birko, Ma’n H. Zawati

**Affiliations:** Centre of Genomics and Policy, McGill University, Montréal, QC, Canada

**Keywords:** clinical trials, gene therapy, health research regulation, health researcher duties, pediatric, stem cells

## Abstract

The pediatric context poses specific challenges to the clinical translation of cell and gene therapies. We conducted a narrative review of literature addressing the responsibilities of researcher-clinicians in the context of clinical trials using cell and gene therapies, with an emphasis on first-in-human trials in minors. The inclusion criteria of our search yielded 16 papers, one of them addressing the exact intersection we were looking at, with the others circling this intersection. Only one of these 16 papers make a link to the wider theoretical debate on the tension between fulfilling the aims of clinical care and the aims of clinical research when conducting clinical trials–what is often referred to as the *similarity vs. difference* debate–and this debate’s connection to discussions of the central concept of the therapeutic misconception. Building on others’ claim that the *similarity vs. difference* framing is too simplistic to capture real-world nuance, we note that the concepts of therapeutic misconception and clinical equipoise both depend on mutually exclusive positions in this debate. We do not argue for jettisoning either of these concepts. Instead, we consider the resulting paradox to be productive. It is productive to integrate discussions of the therapeutic misconception and clinical equipoise into the *similarity* vs. *difference* debate, as the limitations of these discussions become more apparent in conjunction. In other words, researcher-clinicians taking their ethical duties seriously do not need to ‘solve’ these debates, but neither should this free them from engaging with the debates. Additionally, we discuss the legal context of cell and gene therapies in North America, concluding that nothing in the surveyed ethical literature suggests that greater uncertainties inherent in the field of early-phase cell or gene therapy trials justify relaxation of standards around informed consent for cell and gene therapy clinical trials.

## 1 Introduction

Cell and gene therapy research is a significant field, both in terms of its existent and potential medical advances as well as the economic consequences thereof (Bela, 2024; Dowling et al., 2024; Mallapaty, 2025; Birko and Zawati, 2024). Much of the ethical discussion concerns predatory ‘clinics’ selling unapproved interventions to desperate patients. Besides such predatory clinics, conventional translational research in regenerative medicine occurs too. The present article is an overview of what extant literature has to say about the legal and ethical responsibilities of researcher-clinicians running first-in-human pediatric cell or gene therapy clinical trials.

In Canada, clinicians have four duties–the duty to inform, to treat, to follow, and the duty of confidentiality (Canadian Medical Association, 2018; Gouvernement du Québec, 2002). It is often taken for granted that what differentiates the duties of the researcher from those of the clinician engaging in clinical care (if there is indeed something that differentiates it) is the former’s orientation towards society (or the healthcare system, to be more precise) while the clinician’s duties are oriented towards the individual patient (World Medical Association, 2013; Secretariat on Responsible Conduct of Research, 2022). Some scholars adhere to what has come to be called the similarity position, the strong version of which claims that researcher duties cannot conflict with clinician duties, meaning thereby that research must be guided by the duties of clinicians. A weaker version of the similarity position claims that even if clinician duties *can* conflict with those of researchers, the former have priority over the latter. By contrast, the difference position is the one where medical research should not be guided by the duties of clinicians, and a different set of duties is needed to guide it. Furthermore, there are scholars advocating for a ‘middle ground’ position, between the positions of similarity and difference, also called the ‘dual-role’ position, which acknowledges that both sets of obligations make up the dual role of the researcher-clinician (Morain et al., 2023; Hay-Smith et al., 2016). On this position, the researcher and clinician duties are different, but instead of privileging one over the other, it is attempted to reconcile them simultaneously (Gleick, 1987; Strathern, 1991; Mol, 2002; Oluwole, 2014).

The present paper is the result of a hermeneutic interpretive literature review of what it is about first-in-human pediatric cell or gene therapy clinical trials that affects researchers’ duties to potential trial participants (and their parental guardians), placed alongside the wider debate regarding conflicts between clinician duties and researcher duties. The results section summarizes what past literature has had to say on the responsibilities of researcher-clinicians working on clinical trials in contexts combining various combinations of i) pediatric, ii) cell or gene therapies, and iii) phase 1 trials. The discussion section then gets into the relationships between the concepts of therapeutic misconception–a situation wherein “participants mistake research for clinical care” (Bateman-House et al., 2023) – and clinical equipoise–the position where it is unknown whether there is a difference between the therapeutic values of the experimental and the control treatments of the trial–as well as the aforementioned similarity vs. difference debate, noting the theoretical incompatibility of therapeutic misconception and clinical equipoise, due to each making sense only under incompatible theoretical conditions, namely, the difference or the similarity position. The discussion concludes, however, by advocating to not throw the baby out with the bathwater, and proposes that what has been characterized as the resulting “state of incoherence” in the ethics of clinical trials can be seen as a useful point of departure for those wishing to engage with and implement these concepts and theoretical frameworks in clinical trial ethics.

It is imperative to recognize the current dynamic legal background to cell and gene therapies in the USA. There are efforts in some states to pass laws that explicitly contradict federal requirements for cell therapies. Utah has passed a law in 2024, which contains the following: “A healthcare provider whose scope of practice includes the use of stem cell therapy may perform a stem cell therapy that is not approved by the United States Food and Drug Administration, if the healthcare provider provides the patient with the following written notice before performing the therapy” (Utah State Legislature, 2024). Nevada has a similar law passed in 2023 (Nevada State Legislature, 2023). One may speculate that these are a subset of *right-to-try* laws, i.e., laws allowing terminally ill patients access to unapproved therapies. However, neither the Nevada nor the Utah laws are limited to terminally ill patients. Some have identified a recent tendency by “investors, scientists, and academics […] build [ing] momentum [to] bring American political elites on board” with lifting restrictions on unproven therapies (Knight, 2023). One can make the case scientifically and ethically that stem cell treatments *are* different from other non-cell-based drugs, and the whole premise for the present literature review is that it *is* conceivable for the stem cell or gene therapy context to be ethically salient. This is why it is particularly important to note the push, at least in some quarters, to claim that cell or gene therapies differ from conventional drugs in such a way so as to make current regulation of drugs not applicable to them (Lipworth et al., 2021), which, as we will see in what follows, does not bear out in the academic literature surveyed.

## 2 Methods

The research objective of this literature review was to analyze the legal roles and ethical responsibilities of researcher-clinicians in the context of clinical trials using cell or gene therapies, with an emphasis on first-in-human trials in minors. To achieve this research objective, we used an iterative hermeneutic narrative review methodology, which aims to “deepen understanding of complex phenomena rather than produce an encyclopedic inventory of every published source” (Greenhalgh et al., 2023). In our case, since the research objective is quite narrow, we did aim to find every published source that satisfied our criteria.

### 2.1 Inclusion criteria

To have been included, the literature in question must have been all three of the following:

1. Regarding clinical trials;
2. Regarding at least one of the following: cell therapy, gene therapy, Phase I, pediatric; and
3. Addressing researcher-clinicians professional roles/responsibilities/duties/*etc.*

Judgments as to whether a paper satisfies the above criteria, especially criterion 3, are inherently interpretive. Because the hermeneutic approach relies on interpretive engagement with the literature, the elimination of selection bias is not a methodological expectation (Birchley and Ives, 2022).

Articles and book chapters of any methodological design were eligible for inclusion provided they satisfied the above criteria, and were available in the English language.

### 2.2 Hermeneutic interpretive literature search

Hermeneutic narrative reviews are often used when the potentially relevant literature resulting from database searches is too vast to meaningfully examine in depth. In our case, the rationale was different, in that database searches using keywords were returning excessively large sets of papers, whose examination took lots of time only to yield tiny quantities of papers satisfying the inclusion criteria set. This can be explained, at least in part, by the fact that the focus of our review was on a particular group’s professional roles or responsibilities or duties, or *what they ought to do*, all of which can be spoken about without using any of the words used in the above examples or any of their synonyms, causing finding them using keywords and phrases very challenging.

Instead of relying on database searches, we used reference and citation tracking. Beginning with a set of papers we were already familiar with from previous work that seemed sufficiently relevant (one of which ended up being included itself) and tracking references and citations yielded a set of 12 papers that satisfied the criteria. An additional paper, which was published a few days prior to these searches but which the tracking had not turned up, was caught because its author was followed on twitter and they had tweeted it out. Later iterations of such searches ended up yielding a further three papers that satisfied the inclusion criteria, for a total of 16. Many of the papers that do not make this final count of 16 were instrumental in our understanding of the issues and figure throughout our review but are not included in the official 16 papers “making the cut”.

Although the search for the literature and its reading and analysis are presented separately in this section, it is a crucial feature of hermeneutic narrative literature reviews that these steps are not conducted separately and linearly, but rather iteratively (in what is referred to as the two intertwined hermeneutic circles) (Boell and Cecez-Kecmanovic, 2014). Hermeneutically understanding a body of literature is “shaped by an understanding of the constituent texts, and […] through the fusing of the reader’s and text’s knowledge of a field, termed a ‘horizon’“. The search, then, consists of iterative cycles of reading and analyzing texts to “progressively includ [e] more texts as the reader’s horizon extends” (Prentice et al., 2022). Normally, the flexibility inherent in this methodology is said to yield a richer, more relevant series of findings, until (thematic) saturation is deemed to have occurred. In our case, again, since the potentially relevant body of literature is not so extensive–due to the pointed nature of the research objectives – (mere) saturation was not sought, instead aiming to catch everything relevant.

### 2.3 Analysis

Through repeated analytic reading of the relevant corpus, a data extraction sheet was generated. The themes populating this data sheet were developed inductively (Clarke and Braun, 2017), with themes being added and refined in subsequent readings of articles. The idea of analytic reading is to critically assess and understand each article on its own merits as it pertains to the research objective, within its own context, as well as also within the emerging understanding of the whole corpus (the *horizon*): “A movement back and forth from individual text to the whole body of text enhance [s] the understanding and interpretation through questioning and reasoning” (van der Wath and Van Wyk, 2020), all while paying attention not only to what is written in each article but also to what is “backgrounded” (Papoutsi et al., 2021), with this background behind each study coming into clearer focus as the horizon is extended. The aim of all this is not the production of an analysis that pretends to be unbiased (since ethical arguments can hardly be unbiased (Birchley and Ives, 2022), but rather the presentation of the different biases backgrounded in the literature and an analysis of how these biases affect normative interpretation.

## 3 Results

Only one of the papers found and included in our review addressed specifically researcher-clinicians’ duties and responsibilities in the context of first-in-human pediatric cell or gene therapy clinical trials. The remaining 15 were papers circling the specific context. Four address Phase I pediatric clinical trials, 3 – Phase I cell therapy trials, six – pediatric clinical trials in general, and 2 – cell therapy trials in general (see Table 1 for details as well as Figure 1 for the flow chart and a Venn diagram of the included papers by context).This section will present the discussions pertaining to the researcher-clinicians’ responsibilities, divided up by our five contexts, just listed above, seeing how each element or set of elements inflects the larger debate regarding the potential tension between the duties of a clinician and those of a researcher on a clinical trial.

**TABLE 1:** Included papers.

Author (year)	Title	Context(s)	Major findings
Bateman-House et al. (2023)	*Somatic gene therapy research in pediatric populations: ethical issues and guidance for operationalizing early phase trials*	Paediatric, phase I, (gene therapy)	A review of the main ethical issues in pediatric research involving investigational gene therapies, specifically those surrounding the current state of knowledge about gtgene therapies’ product-related immunogenicity, toxicity, duration, irreversibility, informed consent/assent, trial design (including the question of who ‘goes first’), participant and caregiver burdens, and equity in diagnosis and access to research opportunities
Benedetti and Marron (2021)	*Ethical challenges in pediatric oncology care and clinical trials*	Paediatric	A review of the major ethical challenges in pediatric cancer clinical trials
Boydell et al. (2012)	*Paediatric physician–researchers: coping with tensions in dual accountability*	Paediatric	Drawing on qualitative interviews, this paper identifies ethical challenges and strategies used by physician–researchers in managing dual roles. It considers whether competing obligations could have both positive and adverse consequences for both physician–researchers and patients
Czoli et al. (2011)	*Accountability and pediatric physician-researchers: are theoretical models compatible with Canadian lived experience?*	Paediatric	Although some physician-researchers make references to something like the weak version of the similarity position, the pediatric-researchers interviewed in this study did not describe their dual roles in a way that tightly mirrors any of the existing theoretical frameworks. We thus conclude that either physician-researchers are in need of better training regarding the nature of the accountability relationships that flow from their dual roles or that models setting out these roles and relationships must be altered to better reflect what we can reasonably expect of physician-researchers in a real-world environment
Eaton et al. (2014)	*Money and morals: ending clinical trials for financial reasons*	Cell therapy	Examination of the ethical and social effects arising from stoppping clinical trials solely for financial reasons (as opposed to scientific or medical reasons)
Hester and Ross (2021)	*“Medical Benefit” and Therapeutic Misconception: The Ethical Conundrum of Phase 1 Pediatric Oncology Research*	Paediatric, phase I	Phase I trials ethically should not be justified on the promise of direct medical benefit to the pediatric participants
Heyder et al. (2020)	*Ethical aspects of translating research with human pluripotent stem cell products into clinical practice: a stakeholder approach*	Cell therapy	Public and patient involvement is a necessary prerequisite for dealing responsibly with high-risk/high-potential clinical research such as stem-cell research
Hug (2020)	*Understanding voluntariness of consent in first-in-human cell therapy trials*	Cell therapy, phase I	This article outlines the multidimensionality of voluntariness and identifies what could reduce voluntariness, especially in first-in-human clinical trials involving cell therapies
Hug and Johansson (2017)	*Challenges to informed consent in first-in-human trials involving novel treatments: A case study of Parkinson’s disease*	Cell therapy, phase I	This paper aims to contribute to a better informed consent process for Parkinson’s patients taking part in first-in-human clinical trials of cell replacement therapies
de Melo-Martin et al. (2020)	*“I Want to Do It, But I Want to Make Sure That I Do It Right.” Views of Patients with Parkinson’s Disease Regarding Early Stem Cell Clinical Trial Participation*	Cell therapy, phase I	Participants expressed skepticism about the immediate impact of stem cell research. Nonetheless, such skepticism often reflected an appropriate consideration of the risks and potential benefits of participating in high-risk clinical trials. Despite their skepticism, participants were eager to learn more about stem cell research and clinical trials processes
Ross (2006)	*Phase I research and the meaning of direct benefit*	Paediatric, phase I	Phase I trials do not offer the prospect of direct benefit and classifying them as if they do fails to acknowledge the moral relevance of the researchers’ intent
Roth-Cline and Nelson (2013)	*Parental permission and child assent in research on children*	Paediatric	Although both parental permission and child assent involve the same components of information sharing, comprehension, and voluntariness, how these three components are understood and operationalized should differ depending on the developmental level of the child. For example, the amount of information that a child must comprehend to provide meaningful and developmentally appropriate child assent (or dissent) should be allowed to vary with the age and maturity of the child
Schupmann et al. (2021)	*Do the potential medical benefits of phase 1 pediatric oncology trials justify the risks? Views of the United States public*	Paediatric, phase I	A majority of the US public regards the potential medical benefits of average phase 1 pediatric oncology trials as justifying the risks. This finding suggests that these trials are ethically appropriate and approvable in patients who have no more effective treatment options. At the same time, a significant minority thought the potential medical benefits do not justify the risks, suggesting these trials should be approved only when they have significant social value
de Vries et al. (2011)	*Ethical issues at the interface of clinical care and research practice in pediatric oncology: a narrative review of parents’ and physicians’ experiences*	Paediatric	The integration of research and care has consequences for the informed consent process, the promotion of the child’s best interests, and the role of the physician (doctor vs. scientist). True consent to research is difficult to achieve due to the complexity of research protocols, emotional stress and parents’ dependency on their child’s physician. Parents’ role is to promote their child’s best interests, also when they are asked to consider enrolling their child in a trial. Parents are almost never in equipoise on trial participation, which leaves them with the agonizing situation of wanting to do what is best for their child, while being fearful of making the wrong decision. Furthermore, a therapeutic misconception endangers correct assessment of participation, making parents inaccurately attribute therapeutic intent to research procedures. Physicians prefer the perspective of a therapist over a researcher. Consequently they may truly believe that in the research setting they promote the child’s best interests, which maintains the existence of a therapeutic misconception between them and parents
Waligora et al. (2018)	*Neither the harm principle nor the best interest standard should be applied to pediatric research*	Paediatric, phase I	Neither the harm principle nor the best interest standard provide adequate oversight for phase I trials in oncology with elevated risk. We propose the secure child standard instead, complemented by additional conditions: Life-threatening disease with exhausted standard therapeutic options, extended child participation in decision-making process, and maximizing social benefit of performing research
Woods et al. (2014)	*Therapeutic misconception: hope, trust and misconception in paediatric research*	Paediatric	Considers the challenge that the therapeutic misconception presents to parents and explores the ethical and legal responsibilities of researchers to ensure an appropriately informed consent: Compatible with a hopeful disposition of parents who consent for the their children whilst avoiding a blind and misleading optimism

**FIGURE 1:**
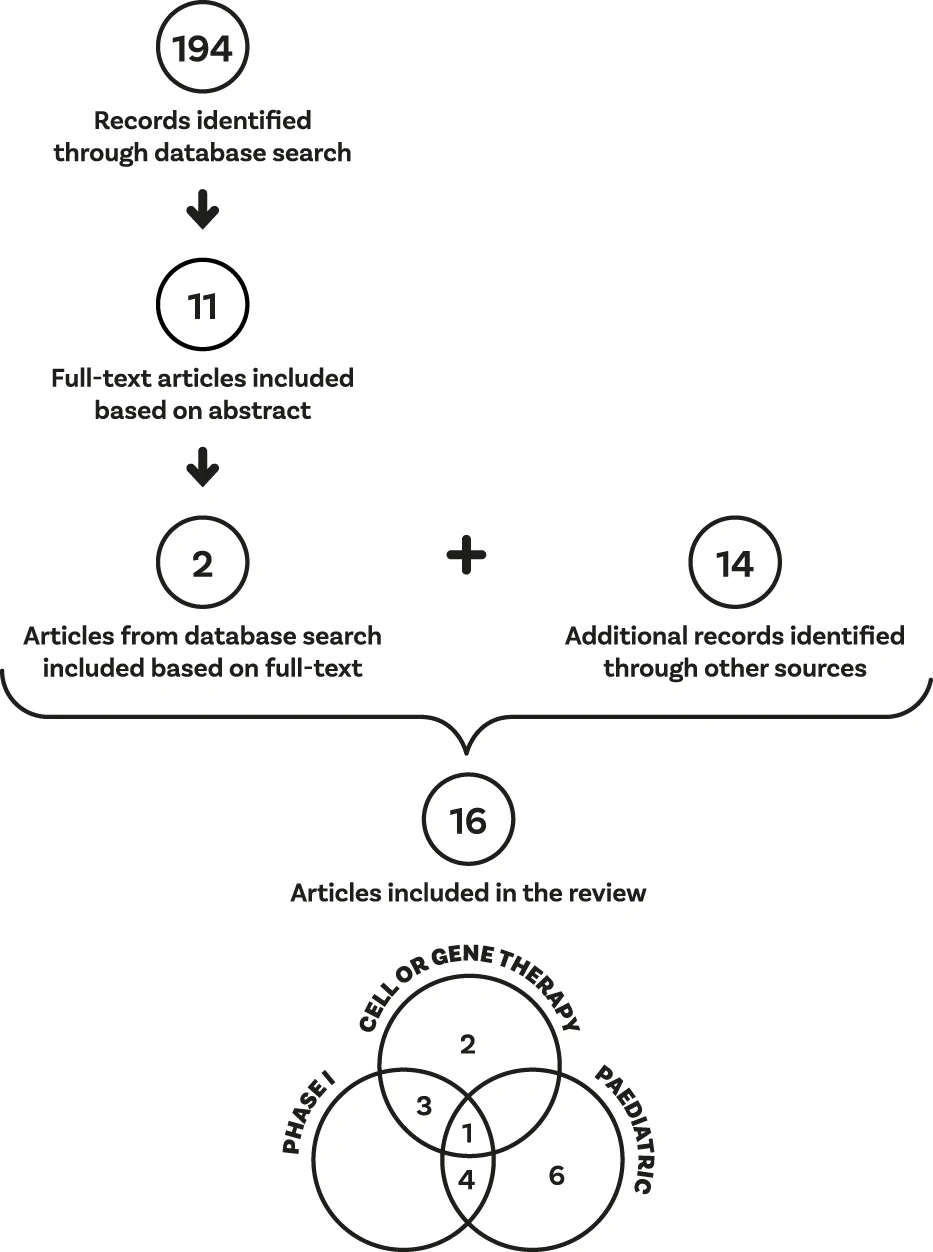
Hermeneutic interpretive literature search flow diagram and included papers by context.

### 3.1 What does the pediatric context add?

The pediatric context significantly affects how informed consent is conceptualized, because instead of the informed consent required from an adult research participant, in the pediatric context, this becomes parental consent (or permission) plus potentially the child’s assent, when deemed capable of providing assent. Roth-Cline and Nelson contend that “[p]arental permission [should be] held to the same standards as informed consent and is required (absent a waiver) for research involving children” (Roth-Cline and Nelson, 2013). They define the child’s assent as being the “child’s affirmative agreement to participate in a clinical investigation” and needing to be more than merely *not objecting* to participate. They go on to state that child assent ought to be conceptualized as evolving “from a choice by young children that is largely dependent on the parent’s decision, to joint decision-making as children mature, to a largely independent decision made by an older adolescent with parental affirmation”, finally evolving to fully independent informed consent when the potential participant is deemed capable of providing such informed consent.

A substantive portion of the discussion in the literature regarding researchers’ duties focuses on the information that must be provided to ensure that consent is properly informed. Before getting into the information the pediatric papers in this review deem important, we first address the question of *who* is a relevant actor in the parental consent and child assent process. Benedetti and Marron try to summarise the *who* by referring to “a triadic relationship, with the child-patient and parents as independent parties in the relationship” (Benedetti and Marron, 2021). Interestingly, the authors seem to omit immediately specifying who the third in the triad is, besides the child and parents, although they do later mention the physician as the “fiduciary of the child-patient, with an obligation to protect and promote the health-related interests of their patient”. Some disagree with Benedetti and Marron’s characterization of the child and the parents as “independent parties”, with Roth-Cline and Nelson explicitly stating that “the meaning and function of child assent should be understood *in the context* of the protection afforded by parental permission” (Roth-Cline and Nelson, 2013) (emphasis added).Woods et al., while “advocat [ing] the right of every child to participate in the decision-making process, to receive appropriately designed information and to be respected in the light of their burgeoning autonomy”, ultimately claim that the law “does not require parents to involve their children in making decisions” and that parental authority carries “the greatest weight” (Woods et al., 2014). Although ‘parents’ is often pluralized when speaking of parental permission, Roth-Cline and Nelson address the question of how many parents need to be involved, making the answer contingent on both the level of risk inherent in the research as well as the existence of *direct benefit* to the participant. Specifically, they claim that the consent of only one parental guardian is “sufficient for research involving minimal risk and greater than minimal risk but presenting the prospect of direct benefit to the child participant”, whereas both parents need to provide consent when the research involves “greater than minimal risk *without* the prospect of direct benefit to the child”.

Regarding the *who* of child assent, Benedetti and Marron announce that there is debate in the scholarly community regarding the age threshold at which child assent becomes a requirement (Benedetti and Marron, 2021) (or even whether age alone can be taken as a sufficient factor in deciding this). Nevertheless, they choose Diekema’s suggestion of 7 years of age as the age after which “most children are developmentally capable of giving assent” (Diekema, 2006). Other scholars argue that older age does not necessarily correlate with greater capacity for assent (Grootens-Wiegers et al., 2017). Roth-Cline and Nelson conclude that assent is necessary in those cases when “failing to require assent (or to respect dissent) in the absence of any prospect of direct benefit shows disrespect for the child’s wishes” (Roth-Cline and Nelson, 2013).The third element of the triad–*who* ascertains parental consent and child assent–is often taken for granted in the literature. Boydell et al. is an exception among our sample, recommending that having a *research coordinator*–who is at ‘arms-length’ from the research–provide information and obtain consent is “an effective means of ‘distancing’ […] from the research and minimizing the potential for coercion or undue influence” (Boydell et al., 2012).

One of the most addressed topics in the literature reviewed is the *what*, as in what information ought to be provided to the parents for consent to be considered informed. There is the view represented by Roth-Cline and Nelson who claim that the pediatric context and the fact that it is the parents making the decision regarding participation on behalf of their child make no difference to the kind of information that is required: “the disclosure of information required for voluntary and informed parental permission is the same as that required for informed consent and includes a discussion of the potential risks, benefits, and alternatives to research participation” (Roth-Cline and Nelson, 2013). Regarding child assent, the same authors outline that the child “should understand why [they] are being asked to participate and what will be [their] experience if [they] decide to participate”. They also specify that some of the elements required for parental permission are *not* required for child assent, namely,: “the reasonably foreseeable risks and benefits, and appropriate alternate procedures or courses of treatment”.

De Vries et al. reiterate some of the requirements for informed parental consent from above and provide further detail: “treatment options, but simultaneously the understanding of the distinction between research and therapeutic intent and of difficult research-related concepts, such as randomization, voluntariness and the risk-benefit ratio” (de Vries et al., 2011). When specifically talking about what empirical studies have shown to be inadequately understood by parents, de Vries et al. add to the above “the procedures”, “the duration of participation, their right to withdraw”. De Vries et al. provide a table of “awareness points and recommendations for communication”, a portion of which we reproduce (Table 2). Czoli et al. remind us that in Canada the *Tri-Council Policy Statement 2* “requires that physician-researchers disclose to research subjects/participants that they are dual role actors” (Czoli et al., 2011).

**TABLE 2:** Portion of Table 3 from de Vries et al.: awareness points and recommendations for communicatio**n** (de Vries et al., 2011).

Awareness	Recommendations for communication
Confidentiality and privacy	Provide information about collection of data and measures taken to protect confidentiality and privacy
The potential for a therapeutic misconception -Difference between clinical research and standard care -Potential conflict between research and treatment goals	Clarify how the physician-investigator/patient-subject relationship differs from the traditional physician-patient relationship
	Mention alternatives to research participation explicitly
	Discuss clinical and parental equipoise
	Mention voluntariness
	Indicate research interventions that are solely performed to measure trial outcomes
	Assure freedom to withdraw from the study
	Discuss research participation with subjects *before, during and after* the study
Consent to treatment does not imply consent to [research]	Ask explicit consent with a separate consent form than the consent form for treatment. Ask consent for treatment first, and for research later, preferably by a different person than the treating physician, for example, a research nurse (with the opportunity to consult with the treating physician)

Although the *what*–what information is required–and the *how*–how should information be presented, as well as how the person providing consent should go about their decision–cannot always be teased apart, in the following subsection we present what our sample of the literature has said about the *how*.

Both parental consent and child assent must be provided *freely*, must be communicated *unambiguously*, and must be *continuous* in time–i.e., the person(s) consenting or assenting must be continuously aware of the option to withdraw (Roth-Cline and Nelson, 2013). Benedetti and Marron specify and attempt to justify the parents’ use of the *best interests standard* when deciding on their child’s participation in research (Benedetti and Marron, 2021; de Vries et al., 2011), rather than the substituted judgment standard, which could be more appropriate in other contexts such as proxy decision-making on behalf of an adult deemed ‘unable to decide for themselves’ (often disparagingly referred to as *incompetent* in the literature).To ensure all the above elements of informed consent and child assent, the researcher has several responsibilities the literature mentions.

Woods et al. explicitly state that “it is the researcher’s responsibility to ensure that adequate procedures for informed consent are in place and that these are satisfied through their best endeavours” (Woods et al., 2014). The researcher must present the information required in a way that the parents and child will understand (“developmentally appropriate” when speaking of child assent) (Roth-Cline and Nelson, 2013; Woods et al., 2014). The amount of the information presented must adequately reflect “how much” the children and parents “desire and need” (Roth-Cline and Nelson, 2013). However, merely presenting the information is not sufficient. Roth-Cline and Nelson repeat several times that the level of the parents’ and child’s understanding must be assessed by the researcher (also (Woods et al., 2014). Woods et al. add that the researcher must “explore possible misconceptions” as part of the informed consent process and must ensure that everyone whose consent and assent are required are appropriately “involved” (also (Roth-Cline and Nelson, 2013)). Woods et al. remind us that “allowing for time to deliberate on the implications of participating in research” is necessary.

Boydell et al. raise additional points to consider regarding *how* the researcher must approach the informed consent process, even outside of the researcher-participant conversation itself: “Reflexivity [i.e., the critical self-reflection and self-inspection of one’s biases, including theoretical and methodological preferences] not only involve [s] critical thinking on the part of the physician-researcher, but also include [s] being reflexive with others in the social network, particularly with the research team, colleague physician-researchers, ethicists, and other professionals” (Boydell et al., 2012).

Some authors spend a considerable amount of time addressing the vulnerabilities that parents deciding on their child’s participation in research face, and how such vulnerabilities may affect the validity of the consent given (Roth-Cline and Nelson, 2013; Woods et al., 2014; de Vries et al., 2011). Stress, in general, makes the resultant consent *fragile* and potentially “*too* fragile to withstand all of the ethical work [it is] required to do” ((Woods et al., 2014), emphasis added). Moreover, being a parent of a “very sick” child or neonate adds even more vulnerability, and even more so “if the request to consent to research comes close to the diagnosis of a serious condition” (Woods et al., 2014; de Vries et al., 2011). Some parents report feeling “pressure to participate in Phase I studies so as not to be seen as ‘giving up’ on their child” (Woods et al., 2014). Such benefits that accrue mainly to the parents–whereas the risks accrue mainly to the child participant–ought not to figure in the decision (Benedetti and Marron, 2021).

Roth-Cline and Nelson refer to empirical studies querying parents of trial participants retrospectively, summarizing that “parents who reported lower voluntariness also perceived more external influence and time pressure, had more concern about the child’s care being negatively affected if they declined, and perceived that they had either too much or not enough information” (Roth-Cline and Nelson, 2013). De Vries et al. also emphasize parents’ fear of “jeopardiz [ing] the relationship with the physician” by refusing their child’s participation in research (de Vries et al., 2011).

If, as Woods et al. advocate, an attitude seeking to “diminish the power differences” “between research regulators, researchers and research participants needs to be fostered” (Woods et al., 2014), and if particular vulnerabilities inherent in our context exacerbate power differences, then researchers’ responsibilities include palliating these vulnerabilities as much as possible. Roth-Cline and Nelson reiterate the utility of “continuous permission” (already introduced above) in palliating these vulnerabilities (Roth-Cline and Nelson, 2013). Moreover, Woods et al. remind us that power imbalances existing outside of the researcher-parent relationship are also relevant, such as those in the parents-child relationship. The authors place the responsibility with the researcher to “ensure that no real or implied coercion is put on those in a dependent relationship”, although it remains unclear how this could realistically be *ensured* by the researcher.

### 3.2 What does the pediatric + phase I context add?

The four papers that add the context of early phase trials to the context of pediatrics center around the discussion of the therapeutic misconception and the related discussion regarding benefits to the participants and their parents.

In the USA, regulations specify that for pediatric research, specifically, whether or not “the prospect of direct benefit” exists is “a critical distinction that influences the level of risk to which subjects can be exposed, the consent mechanisms that are necessary, and whether the study requires local or national review for approval” (Ross, 2006). Ross cites Nancy King’s distinction between three types of research benefits: direct, collateral and aspirational, direct benefit being “benefit arising from receiving the intervention being studied”, collateral benefit being that which “arises from being a subject, even if one does not receive the experimental intervention”, and aspirational benefit being “benefit to society” (King, 2000). Several related debates ensue in the literature and are reproduced in our sample: the debate regarding whether benefit must be direct to justify overriding the pediatric participant’s dissent, and the debate regarding whether phase I clinical trials can ever provide direct benefit.

Hester and Ross acknowledge that “‘medical benefit’ may occur” as a result of participating in a clinical phase I clinical trial, but that what matters more ethically is that such trials “are designed, not to detect any direct therapeutic benefit […] but to evaluate safety in relation to toxicity and side-effect of the drug in question” (Hester and Ross, 2021). Ross had earlier argued similarly that “Phase I trials do not offer the prospect of direct benefit and classifying them as if they do fails to acknowledge the moral relevance of the researchers’ intent” (Ross, 2006). Whether participating in a phase I trial *actually results* in “unintended (collateral) benefits” or not is irrelevant for Ross. Rather, what is morally relevant for Ross is the “researchers’ intent”. Moreover, in 2021, Hester and Ross argued that the uncertainty as to whether participation will result in medical benefit is what disqualifies this uncertain benefit from justifying exposing the participants to greater than minimal risk: “if we could [“say with any confidence what the chances of specifically medical benefit to any particular participant”], we would not need the trial”.

The question as to whether direct benefit will accrue to an early-phase trial participant is relevant not only for deciding what level of risk exposure is justified and what kind of body will have to approve the study, but also because it plays a crucial role in the potential existence of therapeutic misconception. If there is no certainty as to the medical benefits of participating, the participant (or in our pediatric case, the parental guardian(s) making the decision on behalf of the participant) needs to be aware of this lack of certainty, and to know that the purpose of conducting the study–and thereby exposing their child to possible risk–is to try to answer scientific questions regarding toxicity and side effects, in order for their consent to be valid, i.e., for them not to be succumb to the therapeutic misconception. Ross cites Moreno, Caplan, and Wolpe arguing that “[a]ll phase I consent forms should include the phrase, prominently displayed in bold type on the first page, ‘This medical research project is not expected to benefit you’“ (Ross, 2006; Moreno et al., 1998).

However, not everyone agrees with Ross and Hester and Ross (Ross, 2006; Hester and Ross, 2021), and they do not hide this. Ross criticizes the American Society of Clinical Oncology for claiming that “[p]hase I cancer trials can represent a real therapeutic option for some patients who have failed to respond to other treatment or for whom no other therapies exist” (Ross, 2006). They present Kodish as another example of someone “support [ing] viewing Phase I oncology trials as offering the prospect of direct benefit, arguing for a ‘relativistic understanding of prospect of benefit” (Kodish, 2003).

Ross objects to such dissenting views and moreover remarks that “many studies have shown that most subjects enroll in Phase I trials hoping for personal benefit, despite knowing that the study is designed to determine toxicity and despite what is written on the consent form” (Ross, 2006). In addition to participants hoping for personal benefit *despite* consent forms explicitly addressing lack of personal benefit, Ross reports the results of Horng et al. who found that 51% of Phase I consent forms “alluded to the possibility of benefit in a section other than the designated benefit section” (Horng et al., 2002). The false hope resulting from the therapeutic misconception among the parents “may result in parental delay in accepting” their child’s prognosis (Ross, 2006). 59.1% of respondents to Schupmann et al.‘s survey “indicated that it can be appropriate to enroll children for reasons other than a favorable risk-benefit profile. For example, 52% of respondents indicated that it can be appropriate to enroll children in studies that do not offer a favorable risk-benefit ratio because participation offers a way to fight the disease and maintain hope” (Schupmann et al., 2021). This (unwarranted) hope is a perceived benefit that arguably accrues more to the parents rather than the child trial participant.

Nevertheless, if lack of certain medical benefit to participants disqualifies early-phase pediatric trials with more than minimal risk, it would “halt most higher risk nonbeneficial pediatric research”, research with “overwhelming” “social value” (Waligora et al., 2018). Waligora et al. propose then that “the main requirement for pediatric research with elevated risk and low probability of a direct benefit should be the health condition of the child: life-threatening disease with already exhausted standard therapeutic options […] complemented by the prospect of a high social benefit of research and, if possible, by includ [ing] the child’s preferences”.

### 3.3 What does the pediatric + phase I + gene therapy context add?

Bateman-House et al. is the only study satisfying our inclusion criteria that addressed the intersection of all three of our target contexts (Bateman-House et al., 2023). The authors address required elements for informed consent, such as providing information on the nature of medical condition and regarding alternative treatment options, assessing the surrogate decision maker’s “understanding and voluntary agreement”, and using “developmentally appropriate language” when informing older minors. When discussing ongoing consent, i.e., the participants’ “ability to withdraw at any time”, the authors argue that the gene therapy context complicates this supposed ability to withdraw, since once the edited genes are introduced into the body, the effects “cannot be undone” or “turned off”, as compared to traditional drugs, whose concentration in the body decreases once the drug is no longer taken.

Bateman-House et al. also advocate against suggesting any “prospect of direct benefit” as a result of participating in early phase trials so as not to “exacerbate the likelihood of therapeutic misconception”. The authors raise the need to include into informed consent discussions the possibility that “similarly affected relatives are differentially eligible to participate in a trial”, which may result in “potential tensions”, which should be addressed before they arise. The paper additionally addresses researchers’ responsibilities to other healthcare professionals: since the potential participants “receive services from an array of healthcare providers […] all of whom need to understand” the research they would participate in, including “the anticipated effects and risks of these interventions”. Finally, the paper spends some time discussing the possible risk of trial participation precluding participation in future research: “participation in a GT trial often precludes enrollment in other clinical trials, including those with non-GT agents”. The authors do add the caveat that “this consideration may be of less concern in rapidly progressive conditions”.

### 3.4 Cell therapy + phase I context

Hug and Johansson, Hug, and de Melo-Martín et al. address researchers’ responsibilities in the context of early-phase cell therapy trials, i.e., not necessarily pediatric (Hug and Johansson, 2017; Hug, 2020; de Melo-Martín et al., 2020). The responsibilities cluster around the type of information necessary for informed consent as well as how to ensure consent is truly informed.

It is claimed that early-phase trial participants often lack “relevant and adequate information” (Hug, 2020). De Melo-Martín et al. argue that both the stem cell context and the context of clinical trials pose multiple “barriers [to] accessing information”, thereby rendering it the researchers’ (as well as clinicians’, and community groups’) responsibility to provide such information (de Melo-Martín et al., 2020). Hug reports on an empirical study that found that early-phase trial participants were “more likely to be aware of potential risks and their freedom to withdraw at any time” while being “less likely to understand the purpose of the study and that the benefits were uncertain” (Hug, 2020), thereby implicitly placing greater responsibility on researchers to address the latter in order to minimize the potential for therapeutic misconception. In their earlier paper with Johansson, Hug reiterates that cell therapy trials are particular in that one “cannot withdraw from it in the sense of reversing the treatment”, because “injecting cells or making lesions in patients’ brains are irreversible” (Hug and Johansson, 2017).

One responsibility particular to the context is informing participants as to the “sources of cells used”, since “some may hold views on the use of embryonic stem cells or fetal cells, or pig xenogeneic tissue; some may prefer autologous cells, where possible, to donor cells” (Hug and Johansson, 2017). Hug and Johansson ascribe a certain level of responsibility in ascertaining what kind of information each individual participant (or their family) may find important: “researchers could ask the patient about his or her hobbies or try to get a sense of the patient’s priorities in other ways”. Since “obviously, participant information texts cannot be sensitive to individual patients’ goals”, Hug and Johansson emphasize that the responsibility to “take up this issue” – i.e., of individual needs–lies with researchers during verbal discussions.

Hug and Johansson argue that “the real question” [relevant to informed consent] is not about “what types of information should be communicated”, “but rather about what the information-giving process should focus on, and how to assess that the necessary information has been appropriately and accurately communicated”. So, not only are researchers “obligated to accurately communicate to the patient the information relevant to the patient’s decision-making”, but also to make “sure the patient has interpreted the information provided correctly”. The latter responsibility is broken down into both “ensur [ing] that [those deciding on participation] meet the formal requirements for capacity to consent” and “demonstrat [ing] the ability to assess harm-benefit ratios in the proposed study” (Hug and Johansson, 2017). Furthermore, the *early phase + cell therapy* context of the clinical trial suggests that those being recruited “are provided with information ‘precisely at the moment at which they are least able to process it’“, placing additional responsibility on those recruiting to take this vulnerability and the resulting fragility of consent into consideration (Hug, 2020).

Hug places some level of responsibility on the researchers regarding ‘internal’ and ‘external’ influences on potential participants’ decision-making. The author provides examples of ‘internal’ influences as “those caused by health status such as mental illness or dependencies such as alcoholism”. External influences can be those exerted by family and friends, as well as economic conditions (Hug, 2020). The aim of the informed consent process is not to remove the effects of all these influences to arrive at some mythical authentic core identity, as Hug acknowledges. In fact, Hug admits that one cannot expect research ethics guidelines to address such influences, due to the questions raised being “more philosophical in nature and situation-dependent”. However, they do raise the issue in their paper and claim that “it is nevertheless important to raise awareness about those threats to voluntariness and examine how they can affect freedom of decision-making”.

### 3.5 Cell therapy context

Heyder et al. and Eaton et al. are concerned with clinical trials in the cell therapy context (Heyder et al., 2020; Eaton et al., 2014), although Heyder et al. also address the combined cell therapy *and* early-phase context in their paper. Heyder et al. discuss what is necessary “to avoid therapeutic misconceptions or misestimation”: “the explorative nature of the trial should be made explicit and independent counselling provided”. When they discuss the early-phase clinical trial context, they “stress the need for additional information”:

- “the components of the cell product and their origins”;
- “the data collected in preclinical studies”;
- “the relative unpredictability of consequences”;
- “the irreversibility of the process of cell proliferation in the body”;
- “the risk of tumour formation and, possibly, death”.

As Eaton et al.‘s study’s title–*Money and morals: ending clinical trials for financial reasons*–suggests, it focuses on the possibility that clinical trials can be ended purely for financial reasons, not for “scientific or medical” reasons, i.e., “unrelated to safety, efficacy, or feasibility” (Eaton et al., 2014). Stopping (or continuing) clinical trials for any reason will have financial consequences. Eaton et al. focus on the cases when trials are stopped *only* for financial reasons, such as when those conducting the trial find out about another (successful) effort to address the same medical problem, which is forecasted to negatively affect the potential profits of the product being trialed. Another example provided by the authors of purely financial reasons for stopping trials is poor budgetary planning, meaning that the clinical trial in question was poorly budgeted and funding ran out before the trial can be completed. Eaton et al. argue that it is researchers’ (as well as other actors’) responsibility to “ensure that trials are responsibly started”, thereby minimizing the risk of stopping trials for purely financial reasons.

Finally, Heyder et al. address a responsibility nobody else in our sample addressed: the researchers’ responsibility to the donor of the stem cells used in the clinical trial, specifically respecting the right of the donor to decide how their cells can be used. The authors recognize that “because the time, manner and purpose” of future stem cell usage might be unknown when donation occurs, “it is not clear how consent can and should be obtained” (Heyder et al., 2020).

## 4 Discussion

As detailed above, the academic literature captured by our search attributes particular ethical responsibilities to researcher-clinicians conducting clinical trials due to the scientific contexts of i) cell or gene therapies, ii) pediatric, and iii) phase I. While the different combinations of the above three contexts that the papers in our review cover yield overlapping yet non-identical ethical concerns as well as possible ways of mitigating these concerns, there is a common multi-dimensional thread running throughout the discussions. We try to unravel (and to usefully re-entangle) this thread in the discussion section, describing the links between benefits to participating in medical research, the regulatory requirements of conducting such research, and the attendant concepts of therapeutic misconception and clinical equipoise, before noticing the paucity of the *similarity vs. difference* debate in the bioethical literature on researcher responsibilities in our particular research contexts. We highlight what ignoring this debate does to the coherence of the concepts of therapeutic misconception and clinical equipoise. As Miller and Brody have correctly pointed out, this seemingly ‘metaphysical’ omission of the *similarity vs. difference* debate “has left the ethics of clinical trials in a state of incoherence” (Miller and Brody, 2003). We will speculate as to what kinds of epistemologies can lead to such an omission and the taking of one position, the difference position, as the naturalised default. This position, which is presented as the only valid possibility by a subset of the ethical literature, contrasts with what empirical studies conclude is the position implicitly claimed by pediatric researcher-clinicians queried about how they see their roles.

It seems that we end up with more questions and uncertainties than we began with. To be fair, this can be said to be a defining feature of bioethical discussions such as this. Nevertheless, we end on a note that aims at resolution, by way of negating the need to resolve the identified paradoxes. Building on Elizabeth Grosz’ ‘concept’ and Rosi Braidotti’s ‘productive paradox’, we argue that the irreconcilable nature of the central concepts the bioethics literature focuses on may constitute such a ‘productive paradox’ (Grosz, 2010; Braidotti, 2001).

### 4.1 Therapeutic misconception and/or clinical equipoise: ignoring the similarity-difference debate “has left ethics […] in a state of incoherence”

The bioethical literature on clinical trials often centers the concept of direct benefit to a participant in medical research, what it is, whether it exists for a particular study, what its presence or absence entail. There is a burgeoning empirical literature on patients enrolling in early-phase clinical trials because they perceive a possibility of personal benefit (as well as for other reasons), even after being told that the likelihood of personal clinical benefit is very low and it is not being sought by the research team. This empirical literature seeks to understand how such perception of benefit is shaped and discusses whether this constitutes an ethically unacceptable misconception or an understandable way for participants to experience hope (Bergmann et al., 2022; Christopher et al., 2017; Dolly et al., 2016; Escritt et al., 2022; Krutsinger et al., 2024; Locock and Smith, 2011; McCann et al., 2010; Parle et al., 2025; Pentz et al., 2012).

Research benefits have been differentiated into three distinct types: *direct* benefit “arising from receiving the intervention being studies”, *collateral* benefit, which “arises from being a subject, even if one does not receive the experimental intervention” such as a free medical exam, and *aspirational* benefit, which is not a benefit to the participant at all, but rather a benefit to society, which comes about by answering the research question that the study is set up to answer (King, 2000). The reason that the distinction between direct and indirect benefit is claimed to matter is that, e.g., according to the US guidelines on protecting pediatric research subjects (CFR), if the research offers the prospect of direct benefit–i.e., if the “relation of the anticipated benefit to the risk is at least as favorable to the subjects as that presented by available alternative approaches” – then pediatric subjects can be exposed to *greater than minimal risk*, and some argue that consequently, consent mechanisms can be less stringent, as can be the level of review required to approve the study (Ross, 2006).

It is important to pause for a moment to acknowledge important critiques of the technocratic language of risk-benefit calculations used in the literature and in this review. “[A]lthough technical risk-benefit calculations do consider uncertainties, the focus on risks and potential benefits in the ethical analysis of these technologies usually obscures concerns about uncertainties in knowledge and conceals the limits of even the best scientific data” (de Melo-Martín, 2016), thereby obscuring the inherently political nature of risk assessment (Hall, 2023). In other words, the quantifying and measuring language of “relation of the anticipated benefit to the risk [being] at least as favorable … ” pre-supposes an objective generalizable scale or dimension on which different “benefits” and “risks” can be ranked, rather than benefits and risks depicting highly individualized manners of conceptualizing relations of projected futures with how such futures are valued, which may or may not fall on scales that can be comparable by the person concerned. What is referred to as the de-politicization of risk by “risk-management discourses” (Hall, 2023) contributes to creating an artificial separation between technical risk-benefit calculations done by specialists (for their lay subjects) and the individual consent process. This view stands in stark contrast with taking the informed consent process seriously, where it is acknowledged that the risks and benefits cannot be conceived of in a generalized blanket manner for whole populations of people.

The above consideration notwithstanding, whether “direct benefit” is possible in phase I trials has direct bearing on one of the most central ethical concerns related to pediatric phase I trials–the therapeutic misconception, or failing to “appreciate the difference between research and treatment” (Henderson et al., 2007). Ross, who believes that direct benefit in phase I trials is impossible, laments that.

Many studies have shown that most subjects enroll in Phase I trials hoping for personal benefit, despite knowing that the study is designed to determine toxicity and despite what is written on the consent form [although Ross also points out that most consent forms studied *did* mention benefit]. In pediatrics, this may result in parental delay in accepting their child’s terminal prognosis, and may make it harder for parents to acknowledge their child’s suffering if they decide to pursue cure at all cost (Ross, 2006).

Roth-Cline and Nelson concur that “studies in which parental permission was assessed based on recall have generally reported a therapeutic misconception” (Roth-Cline and Nelson, 2013). Rosenstein concluded that researchers are not immune to the therapeutic misconception either (Rosenstein, 2003).

As potential research participants or their guardians often lack access to information on clinical trials (de Melo-Martín et al., 2020), and more so when it comes to cell or gene therapy trials (Heyder et al., 2020), they depend on researchers to provide the relevant information necessary to make their decision. When it comes to phase I trials, the issue is exacerbated still, since even the researchers may commonly lack “relevant and adequate information” (Hug, 2020). Therefore, the research team is responsible for doing their utmost to ensure that their recruited participants avoid the therapeutic misconception in their decision-making. Given that the therapeutic misconception is the “failure to appreciate the difference between research and treatment” (Henderson et al., 2007), addressing it must consist in doing one’s utmost to ensure that potential participants in clinical trials (and their parental guardians) *do* appreciate the difference between research and treatment. To do so, commentators stress the necessity of making explicit “the explorative nature of the trial” and of providing independent counselling (Heyder et al., 2020). In addition, it may be the role of the research community (among others) to address what they refer to as the “collective therapeutic misconception”, or in other words, hyped-up research in society (Woods et al., 2014). On the other hand, some have recently attempted to overthrow the orthodox view, claiming that “great care needs to be taken to address both the increased likelihood of the therapeutic misconception and/or to communicate to patients that they may, in fact, reasonably expect to benefit from research participation.“ (Heynemann et al., 2024) To do so, they propose explaining “how trials are similar to clinical care and what kinds of benefits participants can reasonably expect, while ensuring that these expectations do not become the basis of undue inducement”.

What is of note is that the literature discussing researchers’ roles in pediatric and/or phase I and/or cell or gene therapy clinical trials, emphasizing among other issues the potential for the therapeutic misconception, mostly seems to ignore the highly relevant literature on the debate between the similarity and difference positions (of course, with the notable exception of Czoli and colleagues, who address it head-on) (Czoli et al., 2011). Yet, if there *is* such a thing as the similarity position–which prioritizes the physician’s duties towards the individual participant’s wellbeing–and if it sometimes *can* guide how physician researchers perceive their duties to clinical trial participants, then in these cases, for these researchers, there is no potential for the therapeutic misconception, especially if we concur that what matters ethically is the researcher’s intent. The potential for the therapeutic misconception depends on there being a difference between research and treatment, namely, a difference between the end goals that orient research and treatment. If the ideals guiding research either do not differ from those guiding treatment (strong similarity position),or if they do, then they matter less (weaker similarity position),then the therapeutic misconception cannot obtain. If all health research is also therapy, then believing health research to be therapy is not a *mis*conception.

Similarly, contending with the difference and similarity positions clears up apparent paradoxes related to the concept of clinical equipoise. *Equipoise* refers to the position where it is unknown whether there is a difference between the therapeutic values of the experimental and the control treatments of the trial. The idea behind equipoise is that it is claimed to be an ethically necessary condition for conducting a clinical trial (Freedman, 2017; Miller and Brody, 2003). However, when we remember the similarity and difference positions, the universality of requiring equipoise comes under question. “Clinical equipoise and all other forms of equipoise make sense as a normative requirement for clinical trials only on the assumption that investigators have a therapeutic obligation to the research participants” (Miller and Brody, 2003), i.e., under the similarity position. And under the view that investigators have a therapeutic obligation to research participants, there can be no potential for therapeutic misconception. Simultaneously endorsing the difference position (“reflected in the federal regulatory system and the Belmont Report”) *and* the doctrine of clinical equipoise (which requires that the similarity position hold) “has left the ethics of clinical trials in a state of incoherence” (Miller and Brody, 2003). Put simply, it is not only the case that the “challenges that a lack of parental equipoise and the therapeutic misconception pose may be *very difficult* to overcome” ((de Vries et al., 2011), emphasis added), but that these challenges are theoretically *impossible* to overcome simultaneously.

To put this differently, there seem to be two parallel debates in the literature, one framed as *similarity vs. difference (and beyond)*, and another one, which presents the difference position as the only possible theoretical possibility, making claims such as “[f]rom an ethical point of view, the goals of treatment and research are fundamentally different” or “He [the physician-investigator] serves two different goals: the best interest of the patient and scientific progress (and thereby the best interest of future patients)” (de Vries et al., 2011). We do not claim that the latter debate is intentionally hiding the former one. In fact, it is not surprising to witness at our current conjecture much ethical research framed in highly *Cartesian* terms, taking *organism* to be somehow completely separate from *environment*, or in Freire’s terms “a person [being] merely *in* the world, not *with* the world or with others” (Freire, 2021). Of course, as soon as it is framed like this, it is immediately obvious that organisms are inherently constitutive of environments, especially when speaking of their own environments (Frost, 2016). It is possible that de Vries et al. put their finger on it when they distinguish the present patient from *future* patients. Does our economic setup make it difficult for so many of us to conceive of *future* patients as equal–rather than discounted (Doganova, 2018) – to the present existing patient, who is framed as a basic unit of analysis, an in-dividual?

Miller et al. (1998) addressed the fallacy of positing the difference position as *inherently* correct:

“The fact that the complexity of clinical research does not permit a clean cut between therapeutic and nontherapeutic studies suggests that alternating between a clinical or scientific orientation will not prove satisfactory. The root meaning of “integrity” is wholeness. The professional integrity of physician investigators depends on a coherent moral identity that is proper to the enterprise of clinical research, which is neither medicine nor laboratory science. We need to cultivate a conception of the moral identity of the physician investigator that integrates the roles of the clinician and the scientist without giving predominance to the one or the other (Miller et al., 1998).”

### 4.2 How do pediatric researcher-clinicians see their roles?

Empirical social science complements the picture. Czoli et al. reported on semi-structured interviews conducted with 30 pediatric researcher-clinicians from three major pediatric hospitals across Canada covering “participants’ descriptions of their professional roles related to clinical care and research, compatibility of their dual roles/responsibilities and how conflict is addressed” (Czoli et al., 2011). The authors conclude that researcher-clinicians interviewed “did not describe their dual roles in a way that *tightly* reflects any of the existing theoretical frameworks”. However, if one looks for less *tight* reflections, the authors report that many of those interviewed “could be viewed as being in line with a weaker version of the similarity position”, meaning they see their roles as being *primarily* those of physicians. Consequently, when in line with a weaker similarity position, these researchers cannot be accurately said to be exposing their patients to potential therapeutic misconception, since therapeutic benefit is what is primarily guiding these researchers, and cannot thus be misconceived by the potential participant. Of course, an alternative way of putting this is that the physician-researchers are victims of the therapeutic misconception themselves, as long as they are in line with a similarity position.

### 4.3 Theoretical frameworks as productive paradoxes

There are several possible ‘solutions’ to the problems thus posed, including at least the following four: 1) the theoretical framework of similarity and difference positions is inadequate (this is what Czoli et al. contend) (Czoli et al., 2011), 2) the similarity position is wrong, and the physician-researchers need to understand this and overcome their own therapeutic misconception in order to address their participants’ potential therapeutic misconception, 3) there is no such thing as potential therapeutic misconception (it would be very difficult to convince the wider scholarly community of this solution), or 4) the framework is not exactly inadequate, i.e., the debate does not have to be “resolved”, but is rather meant to serve as a pedagogical tool for researcher-clinicians to contend with in order to fully grapple ethically with the therapeutic misconception.

In other words, following Elizabeth Grosz, the *similarity vs. difference* framework is not a solution to the problem of which researcher-clinicians’ duties take precedence, because this is a problem that has “no solutions–only ways of living with” the problem (Grosz, 2010). Rather than solving the problem, the framework “enable [s] us to surround ourselves with possibilities for being otherwise”.

Using Rosi Braidotti’s concept of a ‘productive paradox’, we argue that the irreconcilable nature of the central concepts the bioethics literature focuses on may constitute a ‘productive paradox’ – one we should “all linger a little longer on […] without rushing headlong into a hasty resolution” (Braidotti, 2001). That is, the paradoxical nature of the relationship between central concepts driving the ethical discussion does not require us to resolve the paradox. Instead, those affected ought to “linger a little longer” on what makes the relationship paradoxical, rather than trying to reconcile the irreconcilable.

### 4.4 Conclusion

Academic discussions of how to ethically conduct early-phase clinical trials take the threat of therapeutic misconception and how it may compromise informed consent very seriously. Our review of the literature of the responsibilities of researcher-clinicians in the context of clinical trials using cell or gene therapies, with an emphasis on first-in-human trials in minors, has demonstrated the paucity of literature on exactly our area of focus–only one paper we found was about all three: phase one clinical trials, pediatric and cell or gene therapies. Nevertheless, there is literature circling the area of focus. This literature on researcher-clinician responsibilities in these contexts emphasizes issues around informed consent, namely, what kind of information ought to be provided to potential participants and/or their parental guardians in order for consent to be sufficiently informed, how the information ought to be provided, and how to ascertain that the information was, indeed, communicated appropriately to and interpreted correctly by the participants and/or their guardians making the decision regarding participation in the clinical trial.

Ensuring the above elements of informed consent in clinical trial participation in our contexts of interest involves exploring potential misconceptions with the people consenting (or refusing). While there are many potential kinds of misconception regarding participating in clinical research (e.g., misestimating the extent of the social value of the research, i.e., the social value misconception (Earl et al., 2025)), the literature we surveyed focused on the therapeutic misconception, the need to address it with potential participants, and *how* to address it. Somewhat surprisingly, we found the ethical literature on researcher responsibilities in particular kinds of clinical trials to be almost completely disconnected from the more theoretical debate between the similarity and difference positions (and beyond). Only one of the 16 papers that met our inclusion criteria referenced the latter debate.

We argued in this paper that if one is to discuss whether researchers’ ethical responsibilities include addressing the therapeutic misconception of participating in clinical trials or requiring that clinical equipoise hold to some extent in order for the trials to be considered ethical, one should be obligated to also address the wider debate regarding whether the concepts of the therapeutic misconception and clinical equipoise make sense, and if so, under which conditions. As we have seen above, if one accepts the framing of the *similarity vs. difference* debate, then claiming that we have to avoid the therapeutic misconception is reasonable only under the difference position, and conversely, that clinical equipoise must hold is reasonable only under the opposite similarity position. If one believes that answering for oneself the debate’s underlying question (*are research and therapy ethically separate or similar?*) is required in order to make use of the concepts of therapeutic misconception and/or clinical equipoise, then doing so seems like a daunting task, indeed. However, if one accepts that there is not necessarily one correct answer to this debate that holds for all researchers in all situations (or even that the question as posed ought to be deconstructed rather than answered), it becomes less daunting.

We argued that, on the one hand, as Czoli and colleagues conclude, the *difference versus similarity* framing is too binary to capture the real-world nuances that researcher-clinicians deal with (Czoli et al., 2011; Oluwole, 2014; Strathern, 1991), and yet, on the other hand, the concepts of therapeutic misconception and clinical equipoise depend on the same simplistic *purification* of research and therapy away from each other (Latour, 2012). Whether or not pragmatically useful concepts replacing therapeutic misconception and clinical equipoise are possible is beyond the scope of this paper. In the meantime, we argue that it is helpful to integrate discussions of the *similarity versus difference* debate into discussions of therapeutic misconception in clinical trials, as the limitations of both of those discussions become more easily apparent in conjunction. In other words, researcher-clinicians that take their ethical responsibilities seriously ought to be exposed to the similarity versus difference debate and encouraged to engage with it in their own research experiences, without feeling like they must find a correct answer to which they must then fit all their subsequent ethical justifications. Although this link is not completely absent in the literature–as reported on above–it ought to be much more present.

We look forward to future scholarship that will expand this discussion of roles and responsibilities to other stakeholders in CGT clinical trials, beyond the researchers. We also look forward to more CGT researchers and other stakeholders taking part in ethical debates concerning clinical and research duties. Upcoming scholarship ought to help practitioners variously reframe how relevant concepts help them think about ethically designing pediatric CGT trials, rather than only providing them with definitive authoritative ‘off-the-shelf’ ethical answers.

Taking the discussion back to the wider context of recently shifting legislation, which may or may not be part of a large-scale effort to create regulatory loopholes, wherein it is attempted to justify the creation of these loopholes by coopting languages of scientific ambiguity and specificity, we want to emphasize that nothing in the extant ethical literature suggests that the greater uncertainties inherent in the field of early-phase cell or gene therapy clinical trials justify any relaxation of current standards around informed consent for clinical trials, in general (Lipworth et al., 2021). On the contrary, and unsurprisingly, the scholars participating in the debates seem to agree that this increased uncertainty justifies stricter adherence to procedures ensuring consent is truly informed. For example, scholars focus our attention on the fact that the cell and gene therapy context, as opposed to traditional pharmaceutical trials, complicates continuous consent, by virtue of the intervention trialed being potentiall irreversible. This complication of continuous consent makes respecting it even more crucial, by emphasizing the potential irreversibility of the procedure with the potential participant’s family, and simultaneously making sure the participant’s family is continuously aware of the option of withdrawing from the clinical trial. Misinterpreting experts’ criticisms of procedures as calls to relax (or even abolish) the said procedures is not unheard of (van den Heuvel et al., 2010; Birko, 2018). Whether such misinterpretations result from malicious intent or mistaken reasoning notwithstanding, it is incumbent upon the research ethics community to pre-emptively address them.
